# Cytokine expression profile in the bone‐anchored hearing system: 12‐week results from a prospective randomized, controlled study

**DOI:** 10.1111/cid.12615

**Published:** 2018-04-27

**Authors:** Tim George Ate Calon, Joost van Tongeren, Omar Omar, Martin Lars Johansson, Robert‐Jan Stokroos

**Affiliations:** ^1^ Department of Otorhinolaryngology, Head and Neck Surgery Maastricht University Medical Center Maastricht The Netherlands; ^2^ Department of Biomaterials, Institute of Clinical Sciences, Sahlgrenska Academy University of Gothenburg Gothenburg Sweden; ^3^ Oticon Medical AB Askim Sweden; ^4^ Department of Otorhinolaryngology, Head and Neck Surgery University Medical Center Utrecht Utrecht The Netherlands

**Keywords:** BAHA, BAHS, bone anchored hearing system, cytokines, Holgers index, inflammation

## Abstract

**Objective:**

To study the effect of implanting the percutaneous bone‐anchored hearing system (BAHS) itself and inflammation of the peri‐abutment skin warrant clarification. In this study, we aimed to acquire further insight into the immune responses related to BAHS surgery and peri‐implant skin inflammation.

**Materials and Methods:**

During surgery and 12 weeks post‐implantation, skin biopsies were obtained. If applicable, additional biopsies were taken during cases of inflammation. The mRNA expression of IL‐1β, IL‐6, IL‐8, TNFα, IL‐17, IL‐10, TGF‐ß, MIP‐1α, MMP‐9, TIMP‐1, COL1α1, VEGF‐A, FGF‐2 TLR‐2, and TLR‐4 was quantified using qRT‐PCR.

**Results:**

Thirty‐five patients agreed to the surgery and 12‐week biopsy. Twenty‐two patients had mRNA of sufficient quality for analysis. Ten were fitted with a BAHS using the minimally invasive Ponto surgery technique. Twelve were fitted with a BAHS using the linear incision technique with soft‐tissue preservation. Five biopsies were obtained during episodes of inflammation. The post‐implantation mRNA expression of IL‐1β (*P =* .002), IL‐8 (*P =* .003), MMP9 (*P =* .005), TIMP‐1 (*P =* .002), and COL1α1 (*P <* .001) was significantly up‐regulated. IL‐6 (*P =* .009) and FGF‐2 (*P* = .004) mRNA expression was significantly down‐regulated after implantation. Within patients, no difference between post‐implantation mRNA expression (at 12 weeks) and when inflammation was observed. Between patients, the expression of IL‐1β (*P =* .015) and IL‐17 (*P =* .02) was higher during cases of inflammation compared with patients who had no inflammation at 12‐week follow‐up.

**Conclusions:**

As part of a randomized, prospective, clinical trial, the present study reports the molecular profile of selected cytokines in the soft tissue around BAHS. Within the limit of this study, the results showed that 12 weeks after BAHS implantation the gene expression of some inflammatory cytokines (IL‐8 and IL‐1β) is still relatively high compared with the baseline, steady‐state, expression. The up‐regulation of anabolic (COL1α1) and tissue‐remodeling (MMP‐9 and TIMP1) genes indicates an ongoing remodeling process after 12 weeks of implantation. The results suggest that IL‐1β, IL‐17, and TNF‐α may be interesting markers associated with inflammation.

## 1 INTRODUCTION

The percutaneous bone‐anchored hearing system (BAHS) has become an established treatment option for patients suffering from various types of hearing impairment. The BAHS consists of a titanium fixture, together with a pre‐mounted skin‐penetrating abutment, to which a sound processor can be attached.^1^ The fixture is implanted in the retro‐auricular temporal bone and relies on osseointegration for anchorage.^2^

A percutaneous prosthesis penetrates the skin, thereby coming into direct contact with the outer environment, and it may elicit a variety of periprosthetic tissue responses, such as inflammation and infection.^3^ In fact, adverse skin reactions, such as skin overgrowth, soft‐tissue reactions, and infection, represent common complications.^4^ Apart from discomfort and morbidity, recurrent episodes of inflammation can limit the use of the sound processor and in some cases lead to the extrusion of the fixture or voluntary implant removal. To assess the soft tissue surrounding the abutment, Holgers index is commonly used in clinical practice and it is frequently described as an endpoint analysis in trials, although questions regarding its validity warrant further study.^5^, ^6^, ^7^

Materials implanted in hard or soft tissue stimulate different cell types. For instance, inflammatory cells produce cytokines, such as interleukin‐1 (IL‐1), IL‐6, IL‐10, and tumor necrosis factor‐α (TNF‐α), which are involved in regulating the immune response and wound healing. It has been hypothesized that several factors affect the biological response to implants; they include the surgical trauma, the shape and chemical characteristics of the material and the host tissue itself.^8^, ^9^ In addition, shear stress concentration in the mobile skin interfacing the rigid abutment may lead to micro‐trauma and cell damage, resulting in a prolonged inflammatory state in the peri‐abutment skin.^10^, ^11^ Bacterial infection has a major impact on inflammation, where the combined presence of an implant and bacteria affects the local immune response, potentially facilitating the establishment of an infection. Bacterial colonization can be expressed as biofilm and possibly intra‐cellular infection^12^ that may contribute to inflammation.^13^ Surgical techniques, bacterial colonization, a foreign‐body response, and specific immune responses are all likely to contribute to the development of adverse soft‐tissue reactions around a percutaneous abutment. The role of each of the contributory factors remains to be clarified.

The inflammatory process around skin‐penetrating titanium implants is characterized the by presence of different types of leukocyte.^14^ Histological and immunohistochemical analyses have revealed large numbers of inflammatory and immunocompetent cells in the area close to the abutment, suggesting that inflammatory reactions are present in the area facing the abutment, even if the skin is not clinically classified as infected 14. Biopsies taken from patients with clinical signs of inflammation demonstrated a larger number of polymorphonuclear leukocytes, B‐lymphocytes, T‐lymphocytes, and macrophages compared with those without clinical signs of inflammation.^14^

Several studies that correlated crevicular fluid cytokines to inflammatory conditions around dental implants, such as peri‐implantitis, a destructive inflammatory process affecting the bone and soft tissues around osseointegrated dental implants, have been published.^15^ In the last review, IL‐1ß and TNF‐α were identified as major cytokines in relation to peri‐implantitis. In contrast to dental implants, our knowledge of the cytokine expression profile in the soft tissue and peri‐abutment fluid surrounding extra‐oral percutaneous implants is very limited. Recently, Lennerås et al described the molecular profile associated with percutaneous femoral prostheses and correlated it to different microbiological and clinical parameters, including a modified Holgers index of the skin surrounding the abutment. Among several detected correlations, the expression of TNF‐α correlated to the presence of *S. aureus* species, whereas the expression of MMP‐8 correlated to polymicrobial detection. Nevertheless, no correlation was observed between the modified Holgers index and any of the analyzed genes.^16^ In the field of BAHS, one study by Grant et al correlated the production of peri‐abutment fluid exudate and its cytokine content to Holgers index, as an indicator of inflammation.^17^ The Holgers index scores correlated to the fluid volume, as well as with the total amounts of IL‐1ß and IL‐8. However, in the latter retrospective studies, the times of retrieval and analysis were relatively random in relation to implantation surgery.

The aims of this prospective clinical study were: (i) to evaluate the molecular profile of factors related to soft‐tissue healing and inflammation at baseline and after 12 weeks of implantation in BAHS patients treated with either a linear incision or minimally invasive Ponto surgery (MIPS) approaches; (ii) to correlate the gene expression to clinical manifestations of soft‐tissue complications, as judged by Holgers index, as well as to local and systemic factors that may affect the early outcomes of BAHS. The gene expression levels of IL‐1β, IL‐6, IL‐8, TNF‐α, IL‐17, IL‐10, transforming growth factor‐beta (TGF‐ß), MIP‐1α, tissue metabolism (MMP‐9, TIMP‐1, collagen type 1 (COL1α1), vascular endothelial growth factor (VEGF)‐A, basic fibroblast growth factor (FGF‐2), Toll‐like receptor (TLR)‐2, and TLR‐4 were determined in peri‐abutment soft‐tissue biopsies by quantitative real‐time PCR (qRT‐PCR).

## 2 MATERIALS AND METHODS

### 2.1 Ethics

This study was performed in accordance with the Dutch legislation on Medical Research Involving Human Subjects Act and with the ethical standards on human experimentation in the Netherlands. The study was conducted in accordance with the Declaration of Helsinki,^18^ approved by the medical ethics committee at the Maastricht University Medical Centre+ (MUMC+) (NL50072.068.14) and registered at http://clinicaltrial.gov (NCT02438618). CONSORT guidelines were followed (Supporting Information Table S1). Monitoring was performed by the sponsor and TFS Develop (Zaltbommel, The Netherlands). The investigators had unrestricted access to all data.

### 2.2 Population

This study is part of a multicenter randomized, controlled trial (RCT). The study protocol has previously been published.^6^ Patients were recruited at the out‐patient ENT department at MUMC+. The inclusion criteria were at least 18 years of age and found to be eligible for unilateral BAHS surgery. The exclusion criteria were: (I) a history of immunosuppressive disease, (II) use of systemic immunosuppressive medication, (III) bilateral BAHS placement, (IV) relevant dermatological disease (eg, psoriasis, severe eczema), (V) participation in other studies, and (VI) when no suitable site for a 4‐mm wide implant was found during surgery. In addition, patients had to agree to voluntary biopsies during surgery and the 12‐week follow‐up. All patients provided written informed consent.

### 2.3 Procedures

Baseline characteristics including gender, age, body mass index, smoking habits, medical history, and medication were obtained in the case report forms. Patients received a Ponto Wide implant with a mounted abutment (Oticon Medical AB, Askim, Sweden). Prior to incision, skin thickness was measured to determine the appropriate abutment length according to the surgical manual.^19^, ^20^ Surgical techniques included the linear incision technique with soft‐tissue preservation^6^, ^21^ or the MIPS technique.^6^, ^22^ An elaborate description is provided in the protocol publication.^6^ Briefly, in the control group, the linear incision technique with soft tissue preservation was performed. Here, a linear retro‐auricular incision was made and a central area of periosteum is removed. The implant site was prepared using a guide drill and countersink drill. The BAHS was placed with 40–50 Ncm insertion torque setting. The incision was then closed with dermal sutures. The abutment was recovered using a 5‐mm skin punch. 6, 19, 21

In the test group, the MIPS technique was performed. Skin and subcutaneous tissue was removed with a 5‐mm punch. Remaining soft tissue was removed with a raspatorium. The MIPS‐cannula was inserted and filled with saline. The implant site was prepared using the specifically designed guide drill and widening drill. After removal of the cannula, the BAHS was placed with 40–50 Ncm insertion torque setting. An installation indicator was used to aid in the estimation of complete insertion.^6^, ^20^, ^23^

During surgery, bone quality was assessed by the surgeon while drilling as very soft, soft, medium, hard or very hard. Post‐surgery, a healing cap with gauze drenched in ointment (Terra‐cortril, Pfizer Laboratories, New York, USA) was placed on the abutment. Subjects were followed for regular follow‐up visits at 9‐days, 3 weeks, 12 weeks, and 1 year post‐surgery. At 9 days post‐surgery, the healing cap was removed. If necessary, local ointment (Terra‐cortril) was used to promote healing. Instructions for aftercare were provided to all subjects. All subjects received an aftercare kit provided by the implant manufacturer (Oticon Medical AB). Subjects were instructed to clean the skin around their abutment each day using plain water and a soft toothbrush. Three weeks post‐surgery, the implant was loaded with a BAHS sound processor (Ponto Plus or Ponto Plus Power, Oticon Medical AB).

The skin‐penetrating abutment and peri‐abutment skin were assessed at regular follow‐up visits and during extra consultations. The Holgers index grading was used: “0 No irritation; 1 Slight redness; 2 Red and slightly moist tissue, no granuloma formation; 3 Reddish and moist, sometimes granulation tissue; 4 Removal of skin‐penetrating implant necessary due to infection.”^5^ Pain scores indicated by the patient, with 0 indicating no pain at all and 10 indicating the worst conceivable pain, were obtained on all visits.

A 5‐mm skin punch located at the implant site, which is removed during surgery, was collected for baseline gene expression. At the twelve‐week follow‐up, a voluntary 1 mm biopsy (Integra, York, USA) was obtained after infiltration with local anesthetics. During cases of soft‐tissue inflammation, defined as a Holgers index of ≥2, between surgery and the 1‐year follow‐up, an additional biopsy was obtained. All the samples were snap frozen in liquid nitrogen and stored at −80°C. High‐resolution photographs (NIKON D800E, NIKON CORPORATION, Tokyo, Japan) with an additional lens (Nikon AF‐S VR Micro‐NIKKOR 105 mm f/2.8G IF‐ED, NIKON CORPORATION, Tokyo, Japan) were obtained prior to each biopsy (surgery, 12‐week follow‐up and cases of inflammation).

### 2.4 RNA extraction and quantitative real‐time polymerase chain reaction

RNA was isolated from the frozen samples using TRI Reagent (Sigma, St. Louis, MO, USA), according to the manufacturer's protocol. The RNA concentration was measured with the DeNovix DS‐11 spectrophotometer. About 750 ng of RNA was used to transcribe to cDNA using the SensiFast cDNA Synthesis Kit, according to the manufacturer's protocol. Primers were obtained for genes related to inflammation (IL‐1β, IL‐6, IL‐8, TNF‐α, IL‐17, IL‐10, TGF‐ß, MIP‐1α), tissue metabolism (MMP‐9, TIMP‐1, COL1α1), vascularization (VEGF)A, FGF‐2), and bacterial infection (TLR‐2, TLR‐4) (Supporting Information Table S2) (Sigma‐Aldrich, St. Louis, Missouri, USA). PCR experiments were performed by a dedicated technician and reported following MIQE guidelines.^24^ To quantify the mRNA expression levels, a qRT‐PCR analysis was performed with a LightCycler480 (Roche) using a three‐step PCR program. Relative gene expression levels were derived using the LinRegPCR (version 2016.1) method and normalized to the geometric average of two reference genes, cyclophylin A (CyloA) and beta‐2‐microglobulin (β2M). Non‐detectable samples were imputed as half the lowest observed threshold. Fold changes were calculated using the delta‐delta CT method.

### 2.5 Statistical analysis

Statistical analyses were performed using R version 3.3.2 (R Foundation for Statistical Computing, Austria). Statistical significance was established at *P* ≤ .05. Gene mRNA expression is presented for all genes. Due to the small sample size and non‐normality of the data, non‐parametric tests were executed. Differences between baseline and the 12‐week follow‐up were compared using Wilcoxon's signed rank test. Differences between surgical techniques were compared at baseline (pre‐surgery) and post‐implantation at the 12‐week follow‐up using the Mann‐Whitney U test. Samples taken during episodes of inflammation were compared with mRNA expression at 12 weeks using Wilcoxon's signed rank test. The post‐implantation 12‐week follow‐up expression of patients who did not experience an episode of inflammation and the post‐implantation 12‐week follow‐up expression of patients who did experience an episode of inflammation during follow‐up were compared using the Mann‐Whitney U test.

In addition, the post‐implantation 12‐week follow‐up expression of patients who did not experience an episode of inflammation from 12 weeks and onward, was compared with gene expression of patients who did experience an episode of inflammation at any time during the follow‐up (baseline—1 year) using the Mann‐Whitney U test.

Pre‐surgical mRNA (baseline) expression was correlated to bone quality, smoking status, diabetes and body mass index (BMI) using Spearman's rank‐order correlation test. Post‐surgical mRNA expression was correlated to pain scores, Holgers index 0 vs 1 scores, smoking status, the presence of diabetes and BMI using Spearman's rank‐order correlation test.

## 3 RESULTS

### 3.1 Population

About 35 of the total of 49 patients participating in the randomized, controlled trial, running from December 2014 to July 2016, agreed to the voluntary 12‐week biopsy. Biopsies from 22 patients had cDNA of sufficient quality to be processed. Seven patients experienced at least one episode of inflammation and five of them agreed to an additional biopsy. Patient characteristics are summarized in Table 1. About 10 patients were fitted with a BAHS using the MIPS technique and 12 patients were fitted with a BAHS using the linear incision technique with soft‐tissue preservation. Skin thickness and abutment length used per surgical group are presented in Table 1. Soft‐tissue outcomes at follow‐up are presented in Table 2.

**Table 1:** Characteristics

**Age** (years)		51.68 (24.44)	
**Gender**			
Male		7 (32%)	
Female		15 (68%)	
**Body mass index** (kg/m^2^)		29.27 (6.80)	
** Smoking**			
Non‐smoker		16 (73%)	
Smoker		6 (27%)	
**Diabetes**		3 (14%)	
**Skin thickness** (mm)		6.5 (1.9)	
**Surgical technique**			
MIPS		10 (45%)	
Linear incision with soft‐tissue preservation		12 (55%)	
	**MIPS** (*n* = 10)	**Linear incision technique** (*n* = 12)
**Skin thickness** (mm)^a^	6.5	6
**Abutment length**		
9 mm	5	7
12 mm	3	4
14 mm	2	1

For continuous variables, the mean (standard deviation) is presented. For categorical variables, the number (%) is presented.

^a^Median is presented.

**Table 2:** Skin outcomes

Holgers index scores at 12 weeks	
Holgers index 0	12 (55%)
Holgers index 1	10 (45%)
Holgers index >1	0 (0%)
**Additional biopsy during inflammation**	5 (23%)

Numbers (%) are presented.

### 3.2 Post‐implantation expression

**Figure 1:**
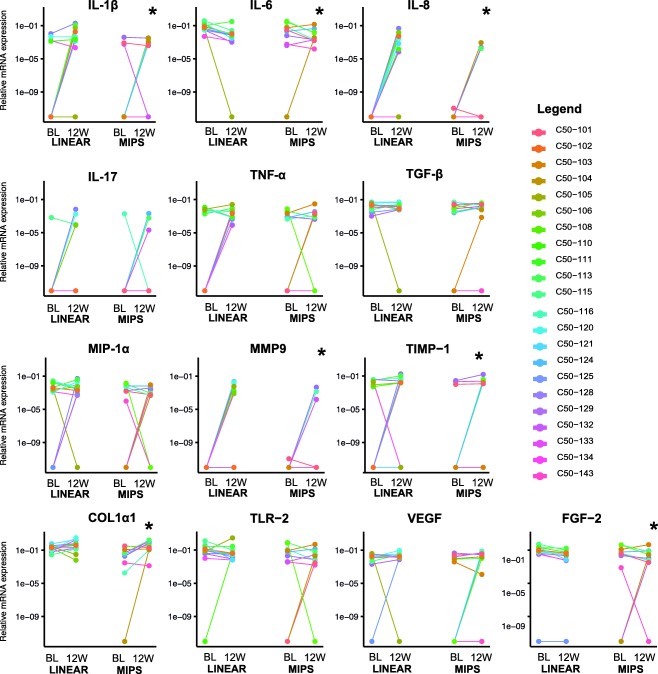
Relative mRNA expression within patients at baseline and 12‐week follow‐up. BL indicates pre‐surgical baseline mRNA expression. The 12w indicates mRNA expression post‐implantation at 12‐week follow‐up visit. LINEAR indicates the linear incision technique with soft‐tissue preservation. MIPS indicates the minimally invasive Ponto surgery technique. Non‐detectable mRNA expression measurements are not shown. In most samples, the mRNA expression of IL‐10 and TLR4 was not detectable and it is therefore not shown. * indicates *P* value ≤.05

The expression of mRNA for the selected cytokines at baseline and the 12‐week follow‐up is shown in Figure 1 and Supporting Information Figure S1. The results for mRNA gene expression at 12 weeks post‐implantation compared with baseline and differences between surgical techniques are presented in Table 3. The variation in mRNA expression at baseline for the different cytokines was high.

**Table 3:** The influence of implantation and surgical technique on cytokine mRNA expression

	Post‐implantation		Surgical technique		
Gene	Fold change	*P* value	MIPS technique	Linear incision technique	*P* value
**IL‐1β**	16.3 (134.5)	.002*	1.2 × 10^−3^ (1.6 × 10^−3^)	2.9 × 10^−3^ (2.4 × 10^−2^)	.06
**IL‐6**	7.3 × 10^−2^ (0.7)	.009*	2.6 × 10^−3^ (1.3 × 10^−2^)	7.1 × 10^−3^ (8.8 × 10^−3^)	.92
**IL‐8**	2.0 (18.9)	.03*	1.0 × 10^−12^ (1.3 × 10^−3^)	4.1 × 10^−4^ (5.7 × 10^−3^)	.06
**TNF‐α**	0.5 (2.9)	.341	9.0 × 10^−4^ (2.2 × 20^−3^)	2.8 × 10^−3^ (3.9 × 10^−3^)	.20
**IL‐17**	1.1 (25.0)	.234	1.0 × 10^−12^ (1.5 × 10^−5^)	1.0 × 10^−12^ (8.3 × 10^−5^)	.84
**TGF‐ß**	1.2 (2.7)	.95	1.4 × 10^−2^ (1.7 × 10^−2^)	1.2 × 10^−2^ (1.8 × 10^−2^)	.95
**MIP‐1α**	0.2 (4.1)	.799	7.4 × 10^−4^ (3.0 × 10^−3^)	2.9 × 10^−3^ (9.2 × 10^−3^)	.16
**MMP‐9**	18.5 (71.5)	.005*	1.0 × 10^−12^ (1.2 × 10^−4^)	8.1 × 10^−4^ (7.8 × 10^−3^)	.11
**TIMP‐1**	1.5 (5.7)	.006*	2.1 × 10^−2^ (2.0 × 10^−2^)	2.1 × 10^−2^ (2.8 × 10^−2^)	1.0
**COL1α1**	1.3 (12.9)	<.001*	0.27 (0.60)	0.42 (0.73)	.58
**FGF‐2**	0.2 (0.4)	.004*	1.9 × 10^−2^ (5.1 × 10^−2^)	3.0 × 10^−2^ (4.0 × 10^−2^)	.64
**VEGF**	0.9(3.8)	.095	2.2 × 10^−2^ (2.6 × 10^−2^)	1.7 × 10^−2^ (1.0 × 10^−2^)	.90
**TLR2**	0.3 (3.1)	.198	1.3 × 10^−2^ (4.9 × 10^−2^)	3.3 × 10^−2^ (4.5 × 10^−2^)	.20

Post‐implantation: the median (interquartile ranges) is presented for fold change post‐implantation compared with baseline. *P* values of relative mRNA expression post‐implantation at 12‐week follow‐up compared with baseline (pre‐surgery) using Wilcoxon's signed rank test are presented. Surgical technique: the median (interquartile ranges) is presented for relative mRNA expression per surgical technique at 12‐week follow‐up. *P* values of relative mRNA expression at 12‐week follow‐up comparing the linear incision with soft‐tissue preservation surgical technique with the MIPS technique using the Mann‐Whitney U test are presented. * indicates *P* value ≤.05.

The analysis revealed that, 12 weeks post‐implantation, a significantly higher expression of the inflammatory markers, IL‐1β and IL‐8, was observed compared with baseline for both surgical techniques, whereas the IL‐6 mRNA expression was significantly down‐regulated. Tissue metabolism markers, MMP9, TIMP‐1, and COL1α1, were significantly up‐regulated 12 weeks post‐implantation. The vascular endothelial growth factor, VEGF, displayed a trend toward increased mRNA expression at 12 weeks. Post‐implantation, the mRNA expression of basic fibroblast growth factor, FGF‐2, was significantly decreased. Compared with baseline, no significant differences were observed for the bacterial marker, TLR‐2.

### 3.3 Surgical technique

**Figure 2:**
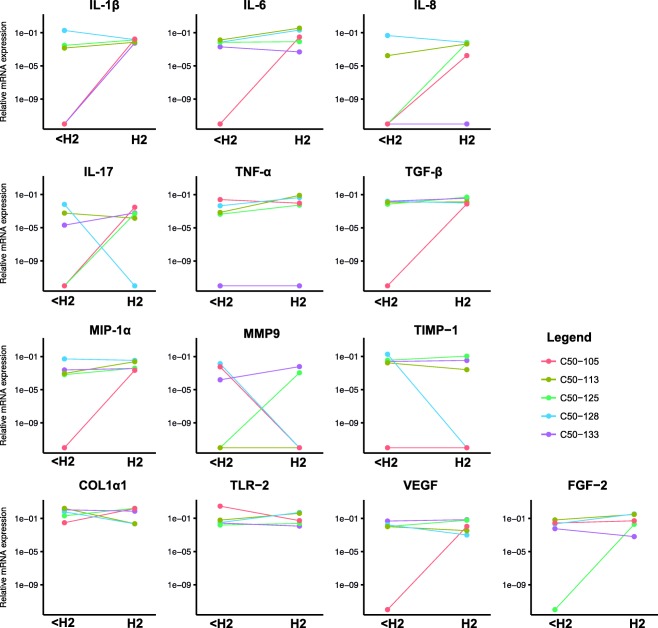
Relative mRNA expression within subjects between 12‐week follow‐up and episodes of inflammation. < H2 indicates relative expression post‐implantation at 12‐week follow‐up with Holgers index 0–1 scores. H2 indicates relative expression during cases of inflammation (Holgers index 2 scores)

The comparative analysis between the two different surgical techniques revealed no significant difference in gene expression in any of the genes at 12 weeks (Table 3). In spite of this, trends toward a higher expression of IL‐8 and IL‐1β were found in the linear incision group compared with the MIPS group (Figure 2; Table 3). No significant differences in gene expression were observed between the groups at baseline (Supporting Information Table S3).

### 3.4 Inflammation

Seven patients experienced an episode of inflammation between 12‐week follow‐up and one year follow‐up. Five of them agreed to an additional biopsy. Of these, one subject had an episode of inflammation 3 months post‐surgery, one 4 months post‐surgery, two 6 months post‐surgery, and one 9 months post‐surgery. The mRNA expression levels at the time of inflammation are presented in Figure 2. The gene expression analysis revealed that, during an episode of inflammation, the pro‐inflammatory cytokines, IL‐1ß, and IL‐17, were significantly up‐regulated compared with patients without an episode of inflammation during follow‐up (Table 4, between patients; Inflammation; Supporting Information Figure S2). For the inflammation samples at the episode of inflammation, TLR‐2, which is related to bacterial recognition, and the inflammatory mediator, TNF‐α, displayed a trend toward higher mRNA expression compared with samples from patients without inflammation. In contrast, in patients experiencing inflammation, no differences in mRNA expression were observed at the time of inflammation compared with the non‐inflamed state at 12 weeks (Table 4, Within patients, Inflammation). Similarly, there were no differences in gene expression at 12 weeks post‐implantation between the 15 patients who did not experience an episode of inflammation during follow‐up compared with the seven patients who did (Table 4, Between patients; Post‐implantation).

**Table 4:** Cytokine mRNA expression during inflammation within and between patients

	Within patients		Between patients	
Gene	Fold change	Inflammation^a^	Post‐implantation^b^	Inflammation^c^
**IL‐1β**	62.3 (15741)	0.188	0.83	0.015*
**IL‐6**	0.9 (3.3)	0.188	0.78	0.23
**IL‐8**	0.02 (0.06)	0.855	1.0	0.16
**TNF‐α**	15.3 (38)	0.361	0.32	*0.05*
**IL‐17**	6.2 (468)	0.813	0.15	0.02*
**TGF‐ß**	3.7 (6.5)	0.188	0.11	0.80
**MIP‐1α**	15.4 (24)	0.438	0.57	*0.10*
**MMP‐9**	17.5 (997)	0.855	0.73	0.96
**TIMP‐1**	1.5 (2.2)	0.855	0.72	0.54
**COL1α1**	4.6 (11)	1.0	0.73	1.0
**FGF‐2**	2.7 (149)	0.125	0.86	0.35
**VEGF**	0.3 (234)	0.438	0.097	0.62
**TLR2**	2.4 (7.2)	1.0	*0.07*	*0.07*

Median (interquartile ranges) fold changes for relative mRNA expression during inflammation compared with baseline are presented. *indicates *P* value ≤.05.

^a^Within‐subject inflammation: post‐implantation mRNA expression at 12 weeks (*n* = 5) is compared with mRNA expression post‐implantation during inflammation (*n* = 5) using Wilcoxon's signed rank test.

^b^Between‐subject post‐implantation (*n* = 22): post‐implantation 12‐week mRNA expression for patients who did not experience inflammation during follow‐up (*n* = 15) is compared with post‐implantation 12‐week mRNA expression for subjects who experienced an episode of inflammation during follow‐up (*n* = 7) using the Mann‐Whitney U test.

^c^Between‐subject Inflammation: mRNA expression during cases of inflammation (*n* = 5) is compared with post‐implantation 12‐week mRNA expression for subjects who did not experience an episode of inflammation during follow‐up (*n* = 15).

### 3.5 Clinical parameters

Multiple exploratory correlations were performed between gene expression and the clinical parameters. Significant correlations with a correlation coefficient of 0.6 or higher are presented in Table 5. The full correlation data sets are included as Supporting Information (Supporting Information Tables S4 and S5). Holgers index 1 compared with Holgers index 0 scores showed a strong negative correlation to the mRNA expression of the pro‐inflammatory cytokine TNF‐α mRNA expression, indicating that Holgers 0 is associated with a higher TNF‐α expression. A strong positive correlation was found for smoking status and post‐implantation MMP‐9 expression, related to tissue metabolism, as well as to extracellular matrix degradation. In addition, a moderate positive correlation was found for post‐implantation IL‐8 mRNA expression and smoking status. Baseline bone quality was moderately associated with a higher mRNA expression of the inflammatory cytokine, IL‐1β, and the vascularization marker, VEGF (Supporting Information Table S4). Further, VEGF mRNA expression at the 12‐week follow‐up was moderately negatively correlated to the presence of diabetes (Supporting Information Table S5).

**Table 5:** Exploratory correlation analyses for mRNA expression and clinical parameters

	Baseline			12 weeks		
Clinical parameter	Gene	*r*_s_	*P* value	Gene	*r*_s_	*P* value
**Holgers index 0 vs. 1**						
				TNF‐α	−.75	<.001
						
**Smoking**						
				IL‐8	.60	.003
				MMP‐9	.85	.04

mRNA expression for moderate to strong statistically significant positive or negative correlations with different clinical parameters analyzed in the patients is presented. The complete output data can be found in Supporting Information Tables S2‐S4.

## 4 DISCUSSION

### 4.1 Summary

To our knowledge, this is the first prospective study to investigate the molecular profile related to inflammation after BAHS implantation. Skin reactions and the local inflammatory gene expression of patients with and without clinical signs of inflammation were compared within and between patients. In addition, the influence of surgical technique was evaluated. The results reveal that, three months after the implantation of a BAHS, there is an up‐regulation of genes related to inflammation (IL‐1β, IL‐8) and tissue remodeling (COL1α1, MMP‐9, and TIMP‐1) within the soft tissue close to the abutment. In contrast, IL‐6 and FGF‐2 decreased significantly. During inflammation (Holgers index ≥ 2), the mRNA expression of IL‐1β, TNF‐α, IL‐17, and TLR‐2 increased. Strong, moderate and weak correlations were found for the post‐implantation mRNA expression of various genes and bone quality, smoking status, or presence of diabetes. No significant differences were found between the surgical techniques with respect to gene expression in the soft‐tissue biopsies.

### 4.2 BAHS implantation

Our results indicate that a continuous state of increased immune activation is present within the soft tissue surrounding the abutment, despite a lack of macroscopic signs of inflammation. This is in line with previous microscopic observations.^14^ In addition, the relatively higher expression of tissue anabolic (COL1α1) and catabolic (MMP‐9 and TIMP‐1) genes at 12 weeks compared with baseline suggests an ongoing remodeling process in the skin surrounding the abutment at this point after implantation. We observed a relatively large variation in mRNA expression between individuals at baseline and post‐implantation. Our results did not show a relation between this variation and future inflammation. We believe that, by presenting individual data per patient, a more accurate presentation of variability is provided, allowing for the visual inspection and interpretation of the complex interplay between the biomaterial and tissue, surgical technique, inflammation, and immune response.

The variability in baseline gene expression may reflect differences in the constitutive molecular activities among the different individuals. Given the possibility that the biopsy may contain different tissue types (skin, subcutaneous adipose tissue, and possibly periosteal components) these may have varied between the individuals and hence contributed to the observed variability in the baseline gene expression. The patients' age and local hygiene could be other factors that may influence the baseline gene expression.

In consonance with the previous BAHS study and dental studies, we found that several inflammation and tissue remodeling related genes were upregulated. 17, 25 Strikingly, IL‐6 and FGF‐2 showed a significant downregulation in mRNA expression. Depending on the micro‐environment IL‐6 can have a pro‐inflammatory or anti‐inflammatory properties with a large inter‐individual variability.^26^ Immune responses in peri‐implant tissue are impaired.^27^, ^28^, ^29^ In some, but not all subjects reduced expression of IL‐6 was observed at 12‐week follow‐up. The decreased expression of IL‐6 might be explained as a direct or indirect reduced immune response. Another possibility could be the difference in the tissue sample volume and/or the tissue and cell types when comparing the baseline full‐length bunch biopsy with the relatively smaller sample punch in the tissue surrounding the abutment after 12 weeks. Here, at least any role for the sample volume can be excluded due to the fact that the relative expression was normalized firstly to the total RNA concentration and secondly to the stable reference genes, which in this case compensate for the variability in the same size/volume.

### 4.3 Inflammation and the Holgers index

The Holgers index is routinely used as one of the major endpoints in BAHS studies.^5^, ^30^ It captures external signs of inflammation, such as moistness or redness. In agreement with the observations of Grant et al, we found that IL‐1β, IL‐17, MIP‐1α, TNF‐α, and TLR‐2 may correlate to inflammation.^17^ In contrast, for Holgers 1 scores, we found negative correlations for IL‐1β, TNF‐α, and TLR‐2 mRNA expression. These contrasting results may indicate that the Holgers index may not necessarily be correlated to gene expression as a continuous scale. The Holgers index was mainly designed for skin reduction techniques. With the shift toward skin preservations techniques, a relatively large area of the soft‐tissue column facing the abutment is not observed. Bearing in mind that the only difference between 0 and 1 is “slight redness,” the full soft‐tissue column facing the abutment may be an important factor which is omitted using the Holgers index for skin preservation techniques. Moreover, the difference between Holgers 0 and Holger 1 scores may only reflect between‐subject variations instead of clinically relevant differences requiring treatment, such as the prescription of topical or oral antibiotics.^7^ Pooling available datasets and studies assessing the inter‐ and intra‐rater reliability of the Holgers index may help in identifying the biological validity and reliability of rating scales for peri‐abutment dermatitis.

### 4.4 Surgical technique

MIPS was designed on the assumption that reduced surgical trauma would lead to improved outcomes.^31^ Due to the less invasive nature of MIPS, we hypothesized that immune responses might be less pronounced. The inflammatory cytokines, IL‐1β and IL‐8, display trends toward lower expression in MIPS compared with the linear incision technique, which may be a confirmation of the hypothesis. Mueller et al observed less inflammation and improved healing outcomes for flapless approaches for dental implants.^32^, ^33^ The limited sample size and quality of cDNA obtained for BAHS warrant further study regarding the influence of surgical technique.

### 4.5 Clinical parameters

Smoking, BMI and diabetes have all been postulated as factors for soft‐tissue reactions or implant loss in BAHS and dental studies.^34^, ^35^, ^36^ Recently, Sayardoust et al showed that smoking is associated with different gene profiles post‐surgery for dental implants.^37^ Here, we observed that, with intact skin, no gene expression differences were observed. However, when the skin integrity is impaired by the BAHS, smoking status increases the expression of genes related to inflammation and tissue remodeling indicating a possible biological relationship for this risk factor. Strong to moderate correlations were found for IL‐8 and MMP‐9 expression. BMI and diabetes might affect the gene expression as well. However, only moderate to weak correlations were found here (Supporting Information Table S5). We found no correlation between pain scores and gene expression for BAHS.

### 4.6 Strengths and limitations

This is the first prospective study to evaluate cytokine expression within the soft tissue around the abutment in BAHS. The influence of both installing the device and inflammation was investigated within and between patients. We were able to correlate cytokine expression to several clinical parameters. On the other hand, this study also has several limitations. This study is a tertiary endpoint of a larger randomized controlled trial comparing surgical techniques. For this sub‐study, no formal sample size calculation was executed. Ideally, this study would have been conducted in a larger set‐up with more time points. The resulting sample size is limited, thereby restricting us to create models that include several factors or performing more elaborate factor analyses. Although, cytokine expression will vary from 12 weeks and onward we assumed that for the overall group expression is relatively stable in order to compare 12‐week expression for subjects without inflammation to the cases of inflammation. In the comparative analysis, we would have preferred to execute all the qRT‐PCR reactions in triplicate. However, the cDNA yield was too low to do this and several patients were excluded due to cDNA yields that were too low. We investigated mRNA expression, which may differ from protein expression. However, to limit this possibility, we used protein coding mRNA transcripts. The location of sampling was close to the BAHS, but it may have differed from patient to patient, due to manipulation and movements. At follow‐up, several patients mentioned that they believed they had experienced more complaints such as crust formation or episodes of inflammation after the 12‐week biopsy. By obtaining a biopsy in the area close to the abutment, a possible source of infection may have been created. In future studies, the use of the periostrip paper as a less invasive alternative for collecting the peri‐abutment fluid should be considered.^17^ A non‐invasive method might increase willingness to provide samples, thereby increasing the sample size and enabling the collection of samples at several time points. In dental implants, bone quality is usually assessed using the classification introduced by Lekholm and Zarb.^38^ This qualification includes a radiographic assessment combined with an tactile assessment performed during drilling. Unfortunately, no such qualification exists for BAHS. Also, radiological assessment of the future implant location is rare. Therefore, in this study we used an explorative bone quality assessment scale based on tactile feedback only.

### 4.7 Perspective

Implant‐associated inflammation and infections represent a challenge in BAHS and other medical devices.^29^ New submerged active and passive bone conduction devices that result in intact skin have been introduced in the last few years.^39^, ^40^ Even though they seem like good alternatives, they may provide too low amplification for some patients. Moreover, these solutions require more invasive surgery, are probably more expensive and have relatively large imaging artifacts of at least 9–10 cm for MRI investigations.^41^ Another approach would be to adjust the surface properties of the percutaneous abutment. Abutments coated with hydroxyapatite that are believed to integrate with the skin have been introduced.^42^ An alternative approach using extra‐smooth abutment surfaces is currently being investigated (NCT02304692). Antibiotic‐releasing, steroid‐releasing and silver‐coated abutments might be possible as well. It is hypothesized that the introduction of skin preservation techniques will help the immune system surrounding the abutment to remain intact, thereby improving immune responses. In addition, the use of punch‐only techniques could reduce skin movements. Immune modulation, abutment properties, surgical damage and reduced skin movements may all contribute to the occurrence of inflammation. The combination of bacterial data, cytokine expression, skin movements, and long‐term follow‐up data may provide additional insights into implantation and inflammation.^6^ Here, we found that implantation itself may already result in a state of continued inflammation.

## 5 CONCLUSIONS

As part of a randomized, prospective, clinical trial, the present study reports the molecular profile of selected cytokines in the soft tissue around BAHS. Within the limit of this study, the results showed that 12 weeks after BAHS implantation the gene expression of some inflammatory cytokines (IL‐8 and IL‐1β) is still relatively high compared with the baseline, steady‐state, expression. The up‐regulation of anabolic (COL1α1) and tissue‐remodeling (MMP‐9 and TIMP1) genes indicates an ongoing remodeling process after 12 weeks of implantation. The results suggest that IL‐1β, IL‐17, and TNF‐α may be interesting markers associated with inflammation.

## CONFLICT OF INTEREST

This study is supported by a research grant from Oticon Medical AB (Askim, Sweden). TC is supported by a research grant from Oticon Medical AB (Askim, Sweden). MJ is a paid employee of Oticon Medical. MJ is supported by the research group of Professor P Thomsen (University of Gothenburg) and the resources provided by the Swedish Research Council (K2015‐52X‐09495‐28‐4), the ALF/LUA Research Grant (ALFGBG‐448851), the IngaBritt and Arne Lundberg Foundation and the Area of Advance Materials of Chalmers and GU Biomaterials within the Strategic Research Area initiative launched by the Swedish Government.

## Supporting information

Additional Supporting Information may be found online in the supporting information tab for this article.

**FIGURE S1** Relative mRNA expression at baseline and 12‐week follow‐up. LINEAR indicates the linear incision technique with soft‐tissue preservation. MIPS indicates the minimally invasive Ponto surgery technique

**FIGURE S2** Relative mRNA expression post‐implantation at 12‐week follow‐up and during episodes of inflammation between subjects. < indicates relative expression post‐implantation at 12‐week follow‐up with Holgers index 0‐1 scores. H2 indicates relative expression during cases of inflammation (Holgers index 2 scores). *indicates *P* value ≤.05. ^ indicates *P* values between .05 and .10

**TABLE S1** CONSORT 2010 checklist of information to include when reporting a randomized trial

**TABLE S2** Primers

**TABLE S3** Cytokine expression at baseline

**TABLE S4** Correlation analysis for baseline cytokine expression

**TABLE S5** Correlation analysis for 12‐week follow‐up cytokine expression
