# Supplementary material for: Cytokine expression profile in the bone‐anchored hearing system: 12‐week results from a prospective randomized, controlled study
Source: Clin Implant Dent Relat Res. 2018 Apr 27;20(4):606–16. doi: 10.1111/cid.12615 (PMC6099213; doi:10.1111/cid.12615)
Supplement: Supplementary file 4 — TABLE S2 Primers [file CID-20-606-s004.docx]

**Table S2: Primers**

| **Generic name** | **Forward primer (5’ 🡪 3’ )** | **Reverse primer (5’ 🡪 3’ )** |  |
| --- | --- | --- | --- |
| IL-1β (interleukin 1, beta) | CTGAGCTCGCCAGTGAAATG | TGTCCATGGCCACAACAACT |  |
| IL-6 (interleukin 6) | ACCCCCAATAAATATAGGACTGGA | TTCTCTTTCGTTCCCGGTGG |  |
| IL-8 (interleukin-8 (IL-8)/ CXCL8 | CCACCGGAAGGAACCATCTC | TTCCTTGGGGTCCAGACAGA |  |
| TNF-α (tumour necrosis factor) | CTGGGCAGGTCTACTTTGGG | CTGGAGGCCCCAGTTTGAAT |  |
| IL-17 (interleukin 17) | AACCGATCCACCTCACCTTG | TCTCTTGCTGGATGGGGACA |  |
| IL-10 (interleukin 10) | ACATCAAGGCGCATGTGAAC | TAGAGTCGCCACCCTGATGT |  |
| TGF-ß (transforming growth factor beta) | GGGCTACCATGCCAACTTCT | GACACAGAGATCCGCAGTCC |  |
| MIP-1α (CCL3 C-C motif chemokine ligand 3) | TCGAGCCCACATTCCGTCAC | GCAGCAAGTGATGCAGAGAAC |  |
| MMP-9 (matrix metallopeptidase 9) | CATCCGGCACCTCTATGGTC | CATCGTCCACCGGACTCAAA |  |
| TIMP-1 (metallopeptidase inhibitor 1) | CATCCGGTTCGTCTACACCC | TCTGCAGTTTGCAGGGGATG |  |
| COL1α1 (collagen, type I, alpha 1) | TGCTCGTGGAAATGATGGTG | CCTCGCTTTCCTTCCTCTCC |  |
| FGF-2 (fibroblast growth factor 2) | CCACCTATAATTGGTCAAAGTGGT | TCATCAGTTACCAGCTCCCC |  |
| VEGF-A (vascular endothelial growth factor A) | CTGTCTAATGCCCTGGAGCC | ACGCGAGTCTGTGTTTTTGC |  |
| TLR2 (toll-like receptor 2) | GTGTTGCAAGCAGGATCCAA | GCAGTGAAAGAGCAATGGGC |  |
| TLR4 (toll-like receptor 4) | GAATGCTAAGGTTGCCGCTT | TTAGGAACCACCTCCACGC |  |
| Beta-2-microglobulin (β2M) | TCCATCCGACATTGAAGTTG | CGGCAGGCATACTCATCTT |  |
| Cyclophylin A (CyloA) | CTCGAATAAGTTTGACTTGTGTTT | CTAGGCATGGGAGGGAACA |  |
