# Supplementary material for: Cytokine expression profile in the bone‐anchored hearing system: 12‐week results from a prospective randomized, controlled study
Source: Clin Implant Dent Relat Res. 2018 Apr 27;20(4):606–16. doi: 10.1111/cid.12615 (PMC6099213; doi:10.1111/cid.12615)
Supplement: Supplementary file 5 — TABLE S3 Cytokine expression at baseline [file CID-20-606-s005.docx]

**Table S3: Cytokine expression at baseline**

| **Gene** | **Surgical technique** | |  |
| --- | --- | --- | --- |
|  | **MIPS technique** | **Linear incision technique** |  |
| **IL-1β** | 1.0*10^-12^ (4.3*10^-4^) | 1.0*10^-12^ (1.4*10^-3^) | 0.66 |
| **IL-6** | 2.5*10^-2^ (6.3*10^-2^) | 6.0*10^-2^ (7.5*10^-2^) | 0.16 |
| **IL-8** | 1.0*10^-12^ (0) | 1.0*10^-12^ (0) | 0.32 |
| **TNF-α** | 5.6*10^-4^ (1.9*10^-3^) | 2.4*10^-3^ (5.5*10^-3^) | 0.43 |
| **IL-17** | 1.0*10^-12^ (0) | 1.0*10^-12^ (0) | 0.90 |
| **IL-10** | NA | NA | NA |
| **TGF-ß** | 9.1*10^-3^ (2.2*10^-2^) | 2.1*10^-2^ (2.2*10^-2^) | 0.28 |
| **MIP-1α** | 1.6*10^-3^ (6.1*10^-3^) | 2.7*10^-3^ (6.3*10^-3^) | 0.69 |
| **MMP-9** | 1.0*10^-12^ (0) | 1.0*10^-12^ (0) | 0.32 |
| **TIMP-1** | 1.0*10^-12^ (7.5*10^-3^) | 6.9*10^-3^ (2.5*10^-2^) | 0.19 |
| **COL1α1** | 3.9*10^-2^ (9.3*10^-2^) | 0.19(0.15) | 0.41 |
| **FGF-2** | 4.0*10^-2^ (0.1) | 7.6*10^-2^ (0.17) | 0.31 |
| **VEGF** | 6.0*10^-3^ (1.4*10^-2^) | 1.6*10^-2^ (1.6*10^-2^) | 0.12 |
| **TLR2** | 4.0*10^-2^ (0.1) | 8.2*10^-2^ (0.13) | 0.34 |
| **TLR4** | NA | NA | NA |
| Median relative expression (interquartile ranges) is presented for relative mRNA expression per surgical technique at baseline.  p-values of relative mRNA expression at 12-week follow-up comparing the linear incision with soft-tissue preservation surgical  technique with the MIPS technique using the Mann-Whitney U test are presented * indicates p-value ≤ 0.05. NA: not applicable | | | |
