# Supplementary material for: Cytokine expression profile in the bone‐anchored hearing system: 12‐week results from a prospective randomized, controlled study
Source: Clin Implant Dent Relat Res. 2018 Apr 27;20(4):606–16. doi: 10.1111/cid.12615 (PMC6099213; doi:10.1111/cid.12615)
Supplement: Supplementary file 6 — TABLE S4 Correlation analysis for baseline cytokine expression [file CID-20-606-s006.docx]

**Table S4: Correlation analysis for baseline cytokine expression**

| **Gene** | **Bone quality** | | **Smoking** | | **Diabetes** | | **BMI** | |
| --- | --- | --- | --- | --- | --- | --- | --- | --- |
|  | r_s_ | **p-value** | r_s_ | **p-value** | r_s_ | **p-value** | r_s_ | **p-value** |
| **IL-1β** | 0.51 | 0.02* | -0.21 | 0.34 | -0.27 | 0.23 | -0.06 | 0.80 |
| **IL-6** | 0.02 | 0.93 | 0.16 | 0.47 | 0.03 | 0.89 | -0.12 | 0.58 |
| **IL-8** | 0.26 | 0.25 | -0.15 | 0.51 | -0.09 | 0.70 | -0.05 | 0.82 |
| **TNF-α** | -0.04 | 0.87 | 0.15 | 0.50 | 0.12 | 0.60 | -0.21 | 0.35 |
| **IL-17** | -0.22 | 0.33 | -0.22 | 0.33 | -0.13 | 0.58 | 0.14 | 0.54 |
| **TGF-ß** | 0.23 | 0.31 | 0.26 | 0.24 | -0.17 | 0.46 | -0.05 | 0.82 |
| **MIP-1α** | 0.10 | 0.65 | -0.07 | 0.76 | 0.07 | 0.74 | -0.13 | 0.57 |
| **MMP-9** | 0.26 | 0.25 | -0.15 | 0.51 | -0.09 | 0.70 | -0.05 | 0.82 |
| **TIMP-1** | 0.10 | 0.66 | 0.01 | 0.97 | -0.13 | 0.58 | -0.07 | 0.76 |
| **COL1α1** | 0.31 | 0.16 | 0.16 | 0.47 | -0.14 | 0.55 | -0.20 | 0.37 |
| **FGF-2** | 0.01 | 0.96 | 0.18 | 0.43 | 0.07 | 0.75 | -0.28 | 0.21 |
| **VEGF** | 0.53 | 0.01* | 0.22 | 0.27 | -0.03 | 0.89 | -0.19 | 0.39 |
| **TLR2** | -0.15 | 0.51 | 0.22 | 0.32 | 0.03 | 0.89 | 0.08 | 0.73 |
| * indicates p-value ≤ 0.05. A positive correlation for bone quality indicates harder bone as assessed during surgery. | | | | | | | | |
