# Supplementary material for: Cytokine expression profile in the bone‐anchored hearing system: 12‐week results from a prospective randomized, controlled study
Source: Clin Implant Dent Relat Res. 2018 Apr 27;20(4):606–16. doi: 10.1111/cid.12615 (PMC6099213; doi:10.1111/cid.12615)
Supplement: Supplementary file 7 — TABLE S5 Correlation analysis for 12‐week follow‐up cytokine expression [file CID-20-606-s007.docx]

| **Gene** | **Pain scores** | | **Bone quality** | | **Diabetes** | | **BMI** | | **Holgers 0 vs Holgers 1** | | **Smoking** | |
| --- | --- | --- | --- | --- | --- | --- | --- | --- | --- | --- | --- | --- |
|  | r_s_ | p-value | r_s_ | p-value | r_s_ | p-value | r_s_ | p-value | r_s_ | p-value | r_s_ | p-value |
| **IL-1β** | -0.01 | 0.96 | -0.1 | 0.96 | -0.45 | 0.04* | -0.14 | 0.54 | -0.47 | 0.03* | 0.43 | 0.03* |
| **IL-6** | 0.06 | 0.77 | -0.7 | 0.77 | -0.32 | 0.14 | -0.32 | 0.15 | -0.32 | 0.15 | 0.42 | 0.05* |
| **IL-8** | -0.26 | 0.25 | 0.21 | 0.35 | -0.19 | 0.40 | -0.25 | 0.26 | -24 | 0.29 | 0.60 | 0.003* |
| **TNF-α** | 0.01 | 0.95 | 0.00 | >0.99 | -0.07 | 0.75 | -0.31 | 0.16 | -0.75 | <0.001* | 0.49 | 0.02* |
| **IL-17** | 0.11 | 0.63 | *0.40* | *0.06* | 0.01 | 0.96 | 0.17 | 0.46 | -0.10 | 0.65 | -0.07 | 0.77 |
| **TGF-ß** | 0.04 | 0.88 | 0.12 | 0.61 | -0.43 | <0.05* | -0.04 | 0.88 | 0.06 | 0.79 | 0.04 | 0.87 |
| **MIP-1α** | -0.31 | 0.16 | 0.20 | 0.37 | -0.43 | <0.05* | *-0.36* | *0.10* | -0.33 | 0.13 | 0.45 | 0.03* |
| **MMP-9** | -0.04 | 0.86 | 0.30 | 0.18 | -0.06 | 0.80 | 0.01 | 0.95 | -0.08 | 0.74 | 0.85 | 0.04* |
| **TIMP-1** | 0.04 | 0.85 | -0.16 | 0.49 | -0.37 | 0.09 | *0.39* | *0.07* | -0.29 | 0.20 | -0.13 | 0.56 |
| **COL1α1** | 0.01 | 0.96 | 0.43 | 0.05* | -0.24 | 0.28 | -0.02 | 0.95 | 0.27 | 0.23 | 0.02 | 0.92 |
| **FGF-2** | 0.05 | 0.82 | 0.01 | 0.97 | -0.06 | 0.78 | -0.44 | 0.04* | -0.37 | 0.09 | *0.41* | *0.06* |
| **VEGF** | -0.18 | 0.41 | 0.26 | 0.25 | -0.54 | 0.01 | -0.04 | 0.86 | -0.26 | 0.24 | 0.08 | 0.71 |
| **TLR2** | 0.10 | 0.67 | 0.54 | 0.14 | 0.14 | 0.55 | -0.16 | 0.48 | -0.47 | 0.03* | 0.27 | 0.23 |
| * indicates p-value ≤ 0.05. A positive correlation for bone quality indicates harder bone as assessed during surgery. | | | | | | | | | | | | |

**Table S5: Correlation analysis for 12-week follow-up cytokine expression**
